# Second-Generation Jak2 Inhibitors for Advanced Prostate Cancer: Are We Ready for Clinical Development?

**DOI:** 10.3390/cancers13205204

**Published:** 2021-10-17

**Authors:** Paul Beinhoff, Lavannya Sabharwal, Vindhya Udhane, Cristina Maranto, Peter S. LaViolette, Kenneth M. Jacobsohn, Susan Tsai, Kenneth A. Iczkowski, Liang Wang, William A. Hall, Scott M. Dehm, Deepak Kilari, Marja T. Nevalainen

**Affiliations:** 1 Department of Pathology, Medical College of Wisconsin Cancer Center, Medical College of Wisconsin, Milwaukee, WI 53226, USA; pbeinhoff@mcw.edu (P.B.); lsabharwal@mcw.edu (L.S.); vudhane@mcw.edu (V.U.); cmaranto@mcw.edu (C.M.); kaiczkowski@mcw.edu (K.A.I.); 2 Department of Pharmacology and Toxicology, Medical College of Wisconsin Cancer Center, Medical College of Wisconsin, Milwaukee, WI 53226, USA; 3 Prostate Cancer Center of Excellence at the Medical College of Wisconsin Cancer Center, Medical College of Wisconsin, Milwaukee, WI 53226, USA; plaviole@mcw.edu (P.S.L.); kjacobsohn@mcw.edu (K.M.J.); Liang.Wang@moffitt.org (L.W.); whall@mcw.edu (W.A.H.); dkilari@mcw.edu (D.K.); 4 Department of Radiology, Medical College of Wisconsin Cancer Center, Medical College of Wisconsin, Milwaukee, WI 53226, USA; 5 Department of Urology, Medical College of Wisconsin, Milwaukee, WI 53226, USA; 6 Department of Surgery, Medical College of Wisconsin Cancer Center, Medical College of Wisconsin, Milwaukee, WI 53226, USA; stsai@mcw.edu; 7 Surgical Oncology, Medical College of Wisconsin Cancer Center, Medical College of Wisconsin, Milwaukee, WI 53226, USA; 8 Department of Tumor Biology, H. Lee Moffitt Cancer Center, Tampa, FL 33612, USA; 9 Department of Radiation Oncology, Medical College of Wisconsin, Milwaukee, WI 53226, USA; 10 Masonic Cancer Center, University of Minnesota, Minneapolis, MN 55455, USA; dehm@umn.edu; 11 Department of Laboratory Medicine and Pathology, University of Minnesota, Minneapolis, MN 55455, USA; 12 Division of Hematology and Oncology, Department of Medicine, Medical College of Wisconsin, Milwaukee, WI 53226, USA

**Keywords:** prostate cancer (PC), anti-androgen resistant prostate cancer, castrate-resistant prostate cancer (CRPC), Janus kinase 2 (Jak2), signal transducer and activator of transcription (Stat), solid tumors

## Abstract

**Simple Summary:**

Prostate Cancer (PC) is currently estimated to affect 1 in 9 men and is the second leading cause of cancer in men in the US. While androgen deprivation therapy, which targets the androgen receptor, is one of the front-line therapies for advanced PC and for recurrence of organ-confined PC treated with surgery, lethal castrate-resistant PC develops consistently in patients. PC is a multi-focal cancer with different grade carcinoma areas presenting simultaneously. Jak2-Stat5 signaling pathway has emerged as a potentially highly effective molecular target in PCs with positive areas for activated Stat5 protein. Activated Jak2-Stat5 signaling can be readily targeted by the second-generation Jak2-inhibitors that have been developed for myeloproliferative and autoimmune disorders and hematological malignancies. In this review, we analyze and summarize the Jak2 inhibitors that are currently in preclinical and clinical development.

**Abstract:**

Androgen deprivation therapy (ADT) for metastatic and high-risk prostate cancer (PC) inhibits growth pathways driven by the androgen receptor (AR). Over time, ADT leads to the emergence of lethal castrate-resistant PC (CRPC), which is consistently caused by an acquired ability of tumors to re-activate AR. This has led to the development of second-generation anti-androgens that more effectively antagonize AR, such as enzalutamide (ENZ). However, the resistance of CRPC to ENZ develops rapidly. Studies utilizing preclinical models of PC have established that inhibition of the Jak2-Stat5 signaling leads to extensive PC cell apoptosis and decreased tumor growth. In large clinical cohorts, Jak2-Stat5 activity predicts PC progression and recurrence. Recently, Jak2-Stat5 signaling was demonstrated to induce ENZ-resistant PC growth in preclinical PC models, further emphasizing the importance of Jak2-Stat5 for therapeutic targeting for advanced PC. The discovery of the Jak2V617F somatic mutation in myeloproliferative disorders triggered the rapid development of Jak1/2-specific inhibitors for a variety of myeloproliferative and auto-immune disorders as well as hematological malignancies. Here, we review Jak2 inhibitors targeting the mutated Jak2V617F vs. wild type (WT)-Jak2 that are currently in the development pipeline. Among these 35 compounds with documented Jak2 inhibitory activity, those with potency against WT-Jak2 hold strong potential for advanced PC therapy.

## 1. Introduction

### 1.1. Clinical Problem

Prostate cancer (PC) is currently estimated to affect 1 in 9 men and is the second leading cause of cancer in men in the US [1]. Androgen deprivation therapy (ADT), which targets the androgen receptor (AR), is one of the front-line therapies for systemic PC, which includes metastatic PC and biochemical relapse that has been initially treated surgically or with radiation. ADT, carried out by luteinizing hormone-releasing hormone (LHRH) agonists/antagonists, termed medical castration, reduces the levels of circulating androgens [2,3,4]. In addition to medical castration, anti-androgens, which are competitive antagonists that bind directly to the AR ligand-binding domain, have been used to control PC growth [2,3,4,5,6].

The first-generation anti-androgen bicalutamide competitively inhibits androgen binding to AR [2,3,4,5,6,7] and suppresses the recruitment of AR corepressors in PC [7]. The persistent activity of the AR in PC during bicalutamide treatment led to the development of more potent second-generation anti-androgens, such as enzalutamide (ENZ), which has gained increasing dominance in the clinical space and is currently FDA approved as a monotherapy in both pre- and post-chemotherapy settings [8,9,10,11]. Second-generation anti-androgens, including ENZ, are more effective competitive inhibitors of steroid binding to the androgen binding pocket on the ligand-binding domain of the AR and retains AR more effectively in the cytoplasmic compartment of PC cells [12]. Apalutamide, another second-generation AR antagonist, recently received FDA approval due to the results of the phase III SPARTAN clinical trial, indicating superior metastasis-free survival and reduced symptomatic progression compared to standard of care [13].

Resistance to ADT results in the development of castrate-resistant prostate cancer (CRPC), which occurs in the vast majority of patients with an average onset of 18–24 months after the initiation of medical castration. Castrate-resistant growth of PC is detected with multiple guiding criteria, including imaging, clinical correlation and rising serum levels of prostate-specific antigen (PSA) [14]. In patients with CRPC resulting from medical castration, ENZ is the first therapeutic option to extend the ADT to tissue-level AR inhibition. ENZ, in turn, provides an improvement in patient survival only by 4–6 months due to the rapid development of resistance [9,11,15,16]. Mechanisms underlying PC resistance to ENZ and other anti-androgens are incompletely understood. The currently proposed mechanisms include the emergence of AR splice variants [17,18], glucocorticoid receptor expression [19], a ligand-binding domain mutation F876L in the AR that promotes an antagonist-to-agonist switch of ENZ [20,21] and neuroendocrine differentiation (NE) [22]. Moreover, the emergence of an AR-null, NE-null PC phenotype driven by the MAPK signaling pathway may contribute to ENZ resistance [23]. The activation of the Jak2-Stat5 signaling pathway has been shown to robustly induce viability and growth of PC cells in vitro and in vivo as tumors in mice. In pre-clinical and PC models and clinical PCs, ENZ-induction of a hyperactivated Jak2-Stat5 signaling feed-forward loop was identified as a novel mechanism of ENZ-resistant PC growth [24]. Activated Jak2-Stat5 signaling can be readily targeted by the current Jak2-inhibitors that have been developed for myeloproliferative disorders (MPDs) and hematological malignancies, which will be reviewed here.

### 1.2. Jak1/2-Stat Pathway

#### 1.2.1. Jak2-Stat5 Signaling Pathway

**Figure 1:**
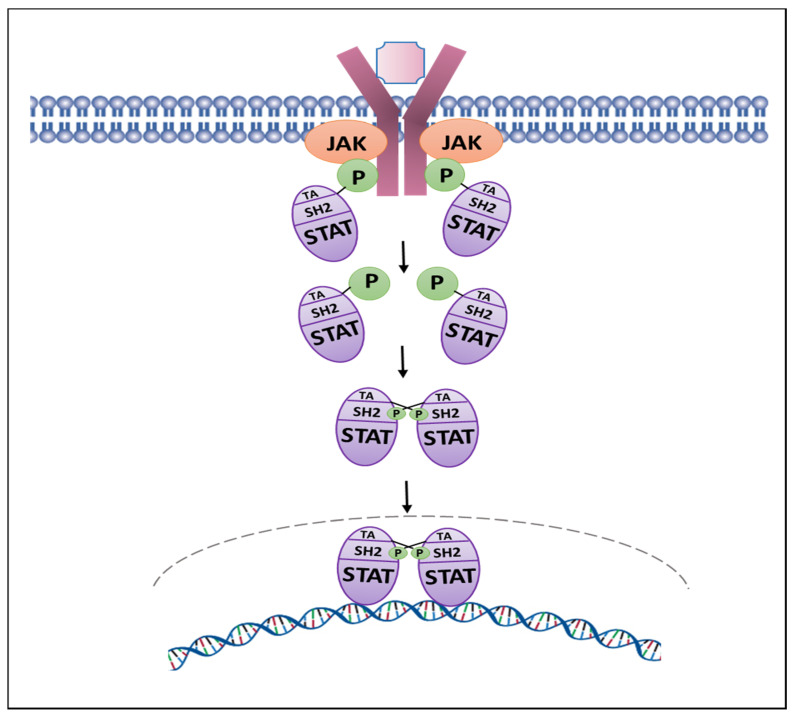
The JAK-STAT pathway. STAT activation is initiated by the binding of an extracellular ligand to trans-membrane receptors, which bring receptor-associated JAKs into close proximity, leading to their activation. The activated JAKs phosphorylate tyrosine residues that serve as docking sites for cytoplasmic STAT transcription factors. STATs bind to JAK through their Src homology 2 (SH2) domain and JAK tyrosine kinases phosphorylate conserved tyrosine residue located between the SH2 domain and the C-terminal transactivation domain (TA) that result in the formation of STAT dimers, which are stabilized by reciprocal phosphotyrosine and SH2 domain interactions. Dimerized STAT proteins translocate to the nucleus to regulate the transcription of their target genes.

Jak2 tyrosine kinase is a member of the Jak family, including Jak1, Jak3 and Tyk2 [25]. Jak2 has seven Jak homology (JH) domains where the JH1 domain represents the kinase domain, JH2 the pseudokinase domain, the JH3-JH4 domains share homology with SH2-domains, and the JH5–JH7 domains represent the FERM domain [25,26]. The binding of cytokines, hormones and growth factors to their specific receptors results in receptor multimerization and recruitment of Jak2 by the cytoplasmic domains of these receptors. The conformational change in Jak2 promoted by receptor binding results in *trans*-autophosphorylation that involves residues Tyr1007 and Tyr1008, resulting in the activation of Jak2 protein [25,26]. Jak2 is the key kinase that phosphorylates Stat5 in PC [27,28,29,30,31]. Stat5 comprises two highly homologous isoforms, Stat5a (94 kDa) and Stat5b (92 kDa) (referred to as Stat5), which are nucleocytoplasmic proteins serving both as cytoplasmic signaling proteins and nuclear transcription factors [32,33,34,35,36]. Upon tyrosine phosphorylation by Jak2, Stat5 forms functional dimers that translocate to the nucleus and bind to specific Stat5 DNA response elements to regulate transcription (Figure 1) [34].

#### 1.2.2. Jak1-Stat3 Signaling Pathway

In PC, Stat3 in PC is activated by Jak1 upon IL-6 stimulation. IL-6 binds to the IL-6 receptor, which is a complex composed of two subunits: IL-6-R alpha (IL-6–specific) and gp130 (shared by IL-6 and related cytokines) [37,38,39]. IL-6 binds to the IL-6R alpha subunit [40], followed by recruitment of the gp130 subunit involved in signal transduction. Association of gp130 with IL-6 and IL-6R alpha forms the high-affinity IL-6 receptor complex leading to activation of the associated Jak1 molecules and phosphorylation of Stat3 on a specific tyrosine residue in the carboxy-terminal domain by a tyrosine kinase [32]. In addition, Stat3 activation is supplemented by phosphorylation of a specific serine residue (S727) [41]. Phosphorylated Stat3 homodimerizes followed by translocation to the nucleus, where it binds to specific Stat3 response elements to regulate transcription [32]. 

#### 1.2.3. Transcription Factor Stat5 Induction of Prostate Cancer Growth, Metastatic Progression and Anti-Androgen Resistance

Stat5 sustains PC cell viability and induces both androgen-sensitive and CRPC growth [30,42,43,44,45,46,47,48]. Conversely, blockade of Stat5 signaling induces apoptotic death of PC cells, suppresses the growth of both xenografted and autochthonous PC tumors and induces cell death in clinical patient-derived PCs ex vivo in explant cultures [24,30,42,43,44,45,46,47,48,49,50,51]. Overexpression of active Stat5, in turn, has been shown to induce proliferation of PC cells in culture and growth of PC tumors in mice [52]. In addition to PC growth promotion, Stat5 induces metastatic progression of PC, as evidenced by increased epithelial-to-mesenchymal transition and stem-like cancer cell properties through induction of Twist1 and BMI1 expression in PC and promotion of metastasis formation in vivo [30,45]. In 30–40% of advanced CRPCs, cytogenetic studies have indicated that the chromosome 17 locus encompassing *STAT5A* and *STAT5B* genes undergoes amplification in subclones of clinical PCs in histological sections leading to increased Stat5 protein levels [52]. High levels of active Stat5 at the time of the initial PC surgery have been shown to predict PC recurrence in three independent cohorts totaling 1035 patients [53,54]. Intriguingly, combined positive status for both Stat5 gene amplification and high Stat5 protein level was independently associated with shorter disease-free survival in univariate analysis and was an independent predictor of PC recurrence in multivariate analysis when compared to the variables of the Cancer of Prostate Risk Assessment Postsurgical nomogram (CAPRA-S) [55]. The predictive role of active Stat5 for clinical PC progression to a lethal CR state [53,54,55] corroborates the data indicating the involvement of Stat5 in PC progression obtained utilizing preclinical PC models.

In PC cells, the Jak2-Stat5 signaling pathway is activated by a number of growth factors and peptide hormones that are present in serum and expressed and secreted locally in PC tissue in an autocrine/paracrine manner [56,57,58,59,60,61,62,63,64,65,66,67,68,69,70,71]. Recently, ENZ-liganded AR was shown to hyperactivate Jak2-Stat5 signaling in CRPC via a mechanism whereby ENZ directly increased activation of both Jak2 and Stat5 and Stat5 further induced Jak2 mRNA and protein levels. In addition to ENZ-induction of Jak2-Stat5 in preclinical models, Stat5 signaling was elevated in clinical PCs from patients treated with ENZ [24]. Further, ENZ-liganded AR was shown to induce a rapid and sustained Jak2 phosphorylation in PC cells through a process involving Jak2-specific phosphatases PTPƐ and SHP2 [24]. Importantly, the ENZ-activated Jak2-Stat5 pathway promoted the growth of PC cells during ENZ treatment and, at the same time, inhibition of Stat5 as a second-line treatment induced extensive death of PC cells surviving ENZ treatment. Most importantly, pharmacological Jak2-Stat5 blockade inhibited CR growth of PC xenograft tumors after ENZ resistance developed and induced further cell death in patient-derived PCs treated with ENZ ex vivo in tumor explant cultures [24]. This work supports a critical role for hyperactive Jak2-Stat5 signaling loop in promoting resistance of PC to ENZ and the need of evaluating the efficacies of Jak2 inhibitors as second-line treatments in advanced PC when ENZ fails. 

#### 1.2.4. Transcription Factor Stat3 and Prostate Cancer Growth

In addition to Stat5a/b, Stat3 has also been implicated in the promotion of growth and PC progression. Stat3 is activated in advanced stages of PC [72,73,74,75,76], accompanied by the interaction with AR in PC cells [77,78,79,80]. In addition, Stat3 has been reported to promote proliferation and inhibit apoptosis in DU145 and LNCaP PC cell lines [72,81,82,83,84]. In preclinical PC models, Stat3 has been shown to increase metastases formation of human PC cells in nude mice, and Stat3 induced PC cell migration in vitro [84,85]. Interleukin-6 (IL-6) is a multifunctional pre-inflammatory cytokine involved in PC pathogenesis [84,86]. IL-6 and IL-6 receptors are expressed in PC [87,88], and IL-6 has been found to be elevated in the sera of patients with CRPC [89,90,91,92,93,94,95]. Moreover, IL-6 is expressed at high levels in the castrate-resistant PC cell lines PC3 and DU145 [94], while the androgen-sensitive PC cell line LNCaP expresses lower IL-6 levels [96]. Many of the current Jak2 inhibitors are also potent inhibitors of Jak1 [84]. Since the IL-6-Jak1-Stat3 pathway is a robust inducer of PC growth and metastasis, simultaneous suppression of Jak1-Stat3 is a desirable off-target effect of the Jak2 inhibitors in therapeutic applications for PC, which would expectedly lead to greater responsiveness of advanced PC to Jak2 inhibitor treatment. 

## 2. Development of Pharmacological Jak2 Inhibitors: Type I and II Inhibitors

An activating V617F mutation in the Jak2 pseudokinase domain causes constitutive activation of Jak2 by disrupting the autoinhibitory action of JH2 [97,98,99,100,101]. The Jak2V617F mutation is found in 90–95% of patients with polycythemia vera (PCV) and 50–60% of patients with essential thrombocytopenia and myelofibrosis (MF) [97,98,99,100,101]. The discovery and identification of Jak2V617F triggered the development of small-molecule Jak2 inhibitors to specifically target hyperactive mutated Jak2. The main platform for the development of Jak2 inhibitors was structure-based drug design, followed by testing the potency in cell-free kinase assays. These Jak2-inhibitors were designed to target both WT-Jak2 and the mutated Jak2V617F in an ATP-competitive manner occupying the ATP-binding pocket of the active conformation of Jak2 [102]. This Type I mode of inhibition stabilizes the kinase in its active conformation [103]. There is numerous Type I Jak2 inhibitors at various stages of development that differ in structure and specificity against WT-Jak2 vs. mutated Jak2V617F, in addition to differing in selectivity for different Jak family members (Table 1, Table 2 and Table 3) [102]. Some of the early first-generation Jak1/2 inhibitors were associated with headache, nausea and neurotoxicity, which were likely due to off-target side-effects and penetration through the blood–brain barrier and led to discontinuation of the clinical development [104]. Later, numerous next-generation Type I Jak2 inhibitors were developed with the decreased capability to cross the blood–brain barrier and improved safety profiles. In contrast, Type II Jak2 inhibitors were designed to bind Jak2 in the inactive conformation, where the inhibitor occupies the ATP binding site and induces a hydrophobic pocket stabilizing the inactive conformation [103]. However, Type II Jak2 inhibitors are not in clinical development. 

### The First-Generation Jak1/2 Inhibitor Ruxolitinib (INCB018424/Jakafi; Incyte, Novartis)

The first-generation pyrrolpyrimidine Jak1/2-inhibitor Ruxolitinib was the first orally available Type I inhibitor of Jak1 and Jak2 to be FDA-approved for MPDs (Table 1, Supplementary Table S1) [102,105,111,112,113]. Preclinical characterization showed that Ruxolitinib had IC50 values for Jak1 and Jak2 of 3.3 nM and 2.8 nM, respectively, in a cell-free assay [111]. In the same biochemical kinase assay, the IC50 of Ruxolitinib for Jak3 was 428 nM [111]. Preclinical MF models indicated high efficacy of Ruxolitinib against cells and tumors expressing Jak2V617F [111]. However, no data have been reported on the potency of Ruxolitinib in any cell-based assays against the WT-Jak2 vs. mutated Jak2V617F [111,114]. 

Safety and early efficacy of Ruxolitinib were evaluated for MF, which indicated that the adverse effects (AEs) centered around diarrhea, fatigue and headache [112,115]. Phase II and III clinical trials (COMFORTI and COMFORT II) testing the efficacy of Ruxolitinib in MPDs indicated significantly positive responses by the reduction in splenomegaly with improved quality of life in patients with MF [116,117,118]. The most notable side effects of Ruxolitinib include thrombocytopenia and anemia in a dose-dependent manner [116,117,118]. Ruxolitinib is primarily metabolized by CYP3A4 [119,120]. Ruxolitinib is currently utilized for the therapy of PCV, with superior results compared to the current standards of therapy [121], and Ruxolitinib is also being investigated for the treatment of psoriasis, alopecia areata and lymphomas. In preclinical models, Ruxolitinib has been evaluated for efficacy in EGFR2-positive breast cancer, squamous cell carcinomas [122,123] and several other solid tumors with variable results [124,125].

Once Stat5 was identified and validated as a therapeutic target protein in PC [30,42,43,44,45,46,47,48], Ruxolitinib was evaluated in an interventional, off-label, single-arm study for its efficacy for treatment of metastatic CRPC (NCT 00638378). This trial was terminated due to the failure to meet the primary outcome of a detectable PSA decline in a significant number of patients. The study was intended to continue for 8 months and evaluate PSA levels monthly. Patients were administered Ruxolitinib 25 mg twice daily in 12 h intervals for 21-day cycles, if well tolerated. Of the 22 patients, 16 reported treatment-related AEs, 9 of which were serious, 13 were grade 3 or 4, and 4 patients discontinued the medication due to AEs. As the primary endpoint was not met, the secondary endpoint was not evaluated (time to progression). 

The lack of efficacy of Ruxolitinib in the PC trial may have been caused by several reasons. First, it is unclear what the potency of Ruxolitinib is against cancer cells expressing WT-Jak2 vs. cells expressing the mutated Jak2V617F. While Jak2 inhibitors have predominantly been developed to target the ATP binding site in the JH1-domain of Jak2 followed by testing in cell-free kinase assays, only sparse data exist for most of the Jak2 inhibitors related to the evaluation of their potency in cell-based assays against WT-Jak2 vs. mutated Jak2V617F. This is relevant because it is known that CRPC cells do not express the mutated Jak2V617F [126]. If Ruxolitinib has its predominant potency against the mutated Jak2V617F, Ruxolitinib is not likely to have efficacy in PC. Second, no selection marker was utilized to identify individual PCs that would respond to Ruxolitinib favorably for accrual to the trial. An example of an effective selection marker would have been the activated Jak2-Stat5 signaling pathway in a pre-treatment biopsy. Third, the patients in this trial were not treated with ENZ or other anti-androgen prior to the accrual. Instead, the patients accrued to this study had received ADT in the form of Gonadotropin-Releasing Hormone (GnRH) agonist (such as Leuprolide or Goserelin), or they underwent a bilateral orchiectomy. Based on the recent finding of ENZ-induction of Jak2-Stat5 pathway activation in PC [24], the PCs of the patients accrued to this trial may have displayed variable activation levels of the Jak2-Stat5 signaling pathway in PC. In conclusion, it would be important to evaluate the efficacy of Ruxolitinib in ENZ-resistant PC with a selection of patients for accrual based on a positive Jak2-Stat5 activation status in PC.

## 3. Next-Generation Type I Jak2-Inhibitors with FDA-Approval or in Clinical Development

### 3.1. Fedratinib (TG101348/SAR302503/Inrebic; TargeGen, Celgene, Bristol Myers Squibb) 

Dianilinopyrimidine Fedratinib is an oral, highly selective FDA-approved (in 2019) Jak2 inhibitor for intermediate/high-risk MF (Table 1, Supplementary Table S1) [127]. Cell-free kinase assays indicate an IC50 of 3 nM against both WT-Jak2 and Jak2V617F with 35-fold selectivity over Jak1 and 334-fold selectivity over Jak3 in a cell-free kinase assay [128]. While the Jak2 inhibitory activity of Fedratinib in cell-based assays has been mostly tested in models expressing Jak2V617F [127,128,129,130], robust evidence has also been presented on the potency of Fedratinib in suppressing WT-Jak2 in cell-based assays [131]. In addition, in preclinical MPD models, Fedratinib effectively inhibited proliferation of both Jak2V617F and FLT3-ITD driven cell lines with a reduction in Stat5 phosphorylation [127,128,129,130]. The safety of Fedratinib was tested in patients with primary or secondary MF in two separate trials, indicating that the main dose-limiting toxicity was an asymptomatic increase in serum amylase levels [132,133]. Other AEs included nausea, vomiting, diarrhea, anemia and thrombocythemia [132,133]. Fedratinib is metabolized by CYP3A4 [134]. Currently, Fedratinib is FDA-approved for MF based on phase II and III trials (JAKARTA), after the data indicated significant reductions of both splenomegaly and disease burden in post-Ruxolitinib setting in patients with MF [135,136]. Fedratinib has a black box warning for encephalopathy that occurs in approximately 1% of patients, leading to a significant delay in the overall development of the drug. Thiamine supplementation has been shown to be correlated with the elimination of encephalopathy as a severe side effect [136]. There are two phase-one clinical trials in unspecified solid tumors (NCT 01585623 and NCT 01836705). 

### 3.2. Pacritinib (SB1518/S-BIO; CTI-BioPharma) 

Pacritinib is a potent macrocyclic pyrimidine-based inhibitor of both Jak2 and FLT3 with minimal potency against Jak1 (Table 1, Supplementary Table S1). The IC50 of Pacritinib against Jak1, Jak2, Jak3 and Tyk2 was 1280 nM, 23 nM, 520 nM and 50 nM, respectively, in a cell-free assay [137]. Most importantly, Pacritinib has been shown to have inhibitory activity against not only Jak2V617F (IC50 19 nM) but also WT-Jak2 (IC50 23 nM) in cell-based assays [137,138,139]. Moreover, Pacritinib was shown to be orally available in mouse models [139] with high efficacy in suppressing Jak2-Stat5 signaling in a subcutaneous human xenograft tumor model [137]. Pacritinib successfully completed phase I and II trials in patients with myeloproliferative neoplasms (MPNs) [140,141,142]. The side-effects of Pacritinib included anemia, changes in hematological parameters (decreased neutrophils, lymphocytes, platelets), diarrhea (grade 1, 2), fatigue and nausea [140,141,142]. Of note, there was minimal impact of Pacritinib on pre-existing anemia or thrombocytopenia in MPN patients. In fact, the majority (58%) of patients in a phase II trial reported symptomatic improvements, and splenic size reduction was observed in 31% of patients up to week 24 of treatment as assessed by MRI [140]. In a randomized clinical trial, Pacritinib was at least twice as effective as the best available therapy in treating MF as measured by splenic volume reduction and symptom scores [143]. The evaluation of the safety of Pacritinib and dosing in a second Phase III trial was launched by CTI-Biopharma and is currently recruiting with a goal of 105 patients with MF after prior failed Ruxolitinib therapy (NCT 03165734). Pacritinib is bound to plasma proteins at a relatively high level in humans and is metabolized by CYP3A4 but is not an inducer of CYP3A4 [144]. Pacritinib has also been investigated in patient-derived xenograft models of glioblastoma multiforme (GBM), which revealed increased survival when combined with adjuvant Temozolomide treatment [145]. Based on the evidence of the potency of Pacritinib against the WT-Jak2 in cell-based assays [137,138,139] and, specifically, in preclinical PC models (unpublished data), Pacritinib is currently being evaluated for safety and efficacy in a single-arm phase II trial (BLAST study) in patients with rising PSA after radical prostatectomy with PSA decline as the primary endpoint (NCT 04635059).

### 3.3. Baricitinib (LY3009104/INCB028050/Olumiant; Eli Lilly) 

Baricitinib is an achiral analogue of Ruxolitinib and a potent, oral Jak1/2 inhibitor with roughly equal inhibitory effects on both kinases in a cell-free assay with IC50 values of 5.9 nM, 5.7 nM, 560 nM and 53 nM for Jak1, Jak2, Jak3 and Tyk2, respectively (Table 1) [146]. Based on testing the potency of Baricitinib in cell-free kinase assays, it has been noted that although both Jak1 and Jak2 were equally inhibited by Baricitinib, there was a 3-fold Jak1 selectivity over Jak2 [147]. No data are available on the efficacy of Baricitinib on WT-Jak2 vs. mutated Jak2V617F in cell-based assays. Currently, Baricitinib is FDA-approved (at 2 mg daily dose) for rheumatoid arthritis (RA) patients failing Tumor Necrosis Factor (TNF) inhibitors [148,149,150]. In addition, there are currently approximately 80 clinical trials testing the safety and efficacy of Baricitinib in other autoimmune diseases and in SARS-CoV-2-patients. The primary AEs of Baricitinib include opportunistic infections, such as herpes zoster, gastro-intestinal (GI) side effects, changes in hematological parameters, malignancy and venous thromboembolism [151,152,153]. No studies have been conducted regarding the efficacy of Baricitinib in solid tumors. 

### 3.4. Momelotinib (CYT 387; Gilead)

Momelotinib is an ATP-competitive phenylaminopyrimidine pan-Jak inhibitor with Jak1 inhibition roughly equal to Jak2 inhibition in addition to suppression of Jak3, JNK1 and CDK2 to a lesser extent (Table 1) [154]. The IC50 values of Momelotinib against Jak1, Jak2 and Jak3 were 11 nM, 18 nM and 155 nM, respectively, in a cell-free kinase assay [154]. Importantly, cell-based assays indicate the inhibitory activity of Momelotinib only against the mutated Jak2V617F [154]. In fact, no data exist on the inhibitory activity of Momelotinib against WT-Jak2 in any cell-based assays. Momelotinib has been shown to be relatively well-tolerated in two phase I/II studies with the key AEs including diarrhea, peripheral neuropathy, thrombocytopenia and dizziness [155,156]. Momelotinib has been shown to improve splenomegaly in MF patients in several different clinical trials [157,158,159,160]. When compared to other standards of therapy, there was a significant reduction in constitutional symptoms as well as a decreased rate of anemia. It was noted, however, that the anemia and splenomegaly improved to a lesser extent in the patients expressing WT-Jak2 compared to patients that were Jak2V617F positive. There are currently 14 clinical trials primarily investigating the use of Momelotinib in MF and PCV. Four of the clinical trials have progressed to phase III, two of which have been completed (NCT 02101268, NCT 01969838). MOMENTUM is a phase III trial in the recruiting phase, aimed to improve the outcome of anemic MF patients with previously ineffective responses to other Jak-inhibitor treatments (NCT 04173494). There are no completed clinical trials testing the efficacy of Momelotinib in solid tumors. A phase Ib trial investigating the efficacy of Momelotinib in non-small cell lung cancer (NSCLC) with adjuvant Trametinib treatment showed no benefit versus monotherapy with Trametinib [161]. Another clinical trial found no clinical benefit of Momelotinib in NSCLC but instead reported increased toxicity when given concomitantly with Erlotinib therapy [162]. Multiple other PCV and pancreatic ductal adenocarcinoma (PDAC) studies have been terminated (NCT 02101021, NCT 01998828, NCT 02244489). 

### 3.5. Gandotinib (LY2784544; Eli Lilly)

Gandotinib (imidazopyridazine aminopyrazole) is an ATP-competitive Jak2 inhibitor currently intended for the therapy of Jak2V617F-positive MPNs (Table 1). Gandotinib was discovered and characterized using a Jak2-inhibition screening assay in parallel with biochemical and cell-based assays [163]. Gandotinib was shown to potently inhibit Jak2V617F-driven signaling in Ba/F3 cells with IC50 of 20 nM, while the IC50 against IL3-stimulated WT-Jak2 in a cell-based assay was only 1183 nM [163]. Gandotinib had no effect on erythroid progenitor cells, reticulocytes or platelets in mice but was shown to significantly reduce BaF3-Jak2V617F tumor burden in the Jak2V617F-induced MPN model. A phase I safety trial indicated that the maximum-tolerated dose of Gandotinib was 120 mg daily based on dose-limiting toxicities of blood creatinine increase or hyperuricemia at higher doses [164]. The most common treatment-emergent AEs, which were also confirmed in a phase II trial [165], were diarrhea, anemia, thrombocythemia and fatigue. The overall response rates were 95%, 90.5% and 9.1% for Jak2V617F positive PCV, essential thrombocythemia (ET) and MF, respectively. In Jak2V617F negative patients, overall response rates were 44% in ET and 0% in MF, supporting the notion of lesser efficacy of Gandotinib in MPNs expressing WT-Jak2 instead of the Jak2V617F [165]. Gandotinib has been tested in three completed phase I clinical trials in MPDs (NCT 01520220, NCT 01577355, NCT 01134120), with one actively recruiting phase II trial (NCT 01594723). Gandotinib has not been tested for efficacy in solid tumors.

### 3.6. Peficitinib (ASP015K/Smyraf; Astellas)

Peficitinib is an oral pan-Jak inhibitor with IC50 values in a cell-free kinase assay for Jak1, Jak2, Jak3 and Tyk2 as follows: 3.9, 5.0, 0.71 and 4.8 nM, respectively (Table 1) [166]. The primary indication of Peficitinib is currently for RA. In Japanese patients with RA and inadequate response to methotrexate (MTX), Peficitinib demonstrated superiority versus placebo in reducing RA symptoms and suppressing joint destruction (NCT 02305849). While Peficitinib was generally well tolerated, most common AEs include nasopharyngitis (39.7%) and herpes zoster (11.7%) in a safety study conducted in 843 patients [167]. There are currently 34 clinical trials for Peficitinib, three of which have completed phase III in Japan for patients with RA (NCT 01638013, NCT 02308163 and NCT 02305849). Peficitinib is now fully approved in Japan for the treatment of patients with RA and failure to respond to MTX [168]. 

### 3.7. Lestaurtinib (CEP-701; Cephalon, Teva Pharmaceuticals) 

Lestaurtinib is an oral multi-kinase inhibitor with efficacy to suppress FLT3, neurotrophin receptor Trk as well as Jak2 in a cell-free kinase assay with an IC50 of 3 nM, <25 nM and 0.9 nM respectively (Table 1). Lestaurinib has been shown to inhibit phosphorylation of mutated Jak2V617F and, consequently, the phosphorylation of downstream targets such as Stat5 (IC50 10 to 30 nM) and Stat3 in the human erythroleukemia cell line HEL92.1.7 (homozygous for Jak2V617F mutation). Lestaurtinib was developed for treatment of various leukemias and myeloid disorders. Of the 14 documented clinical trials, one trial has progressed into phase III to investigate the potential use of Lestaurtinib as concomitant chemotherapy for acute lymphoblastic leukemia (NCT 00557193). There have been mixed results among completed clinical trials. The CEPHALON 204 trial was unable to document the efficacy of Lestaurtinib in patients with AML, likely due to the complex pharmokinetic profile of Lestaurtinib with excessive binding to plasma proteins [169]. A phase I dose-escalation study indicated similar findings, with incomplete inhibition of Stat5 phosphorylation at lower doses of the drug in MF patients [170]. Importantly, Lestaurtinib was evaluated for efficacy in PC with PSA decline as the primary endpoint. However, the study indicated a high level of binding of Lestaurtinib to serum proteins, which limited penetration and uptake of Lestaurtinib to PC tissue (NCT 00081601). This was evaluated by administration of Lestaurtinib daily (40 mg) for 5 days prior to radical prostatectomy followed by evaluation of Lestaurtinib levels in PC tissue [171]. This study illustrated that the pharmacokinetic analyses of Jak2 inhibitor levels in plasma may not reflect the levels present in solid tumor tissues. 

### 3.8. Tofacitinib (CP-690550/Tasocitinib/Xeljanz; Pfizer) 

Tofacitinib is a first-generation, orally bioavailable pan-Jak inhibitor. Tofacitinib was mainly developed for use as an immunosuppressant for organ transplantation and possibly for the treatment of autoimmune diseases (Table 1). Tofacitinib suppresses the Jak family members at IC50s as follows: Jak1 (112 nM), Jak2 (20 nM) and Jak3 (1 nM), in a cell-free kinase assay [172]. In addition, Tofacitinib effectively suppresses common γ-chain cytokines involving IL-2, IL-4, IL-15 and IL-21. Furthermore, Tofacitinib suppresses the signaling by IFN-γ, IL-6, and to a lesser extent IL-12 and IL-23 via Jak1 and Jak2 inhibition. Consequently, Tofacitinib influences the differentiation of CD4+ T helper cells and limits Th17 cells [173]. A study in the PC-3 PC cell line, which has a CRPC phenotype, demonstrated that Tofacitinib inhibited IL-7-dependent phosphorylation of Stat5 and IL-7-induced invasion of PC cells [174]. Tofacitinib has been explored in multiple phase II and phase III clinical trials for efficacy in ulcerative colitis, RA, Down syndrome, alopecia areata, atopic dermatitis, hidradenitis suppurativa vitiligo and psoriasis. The most common side effects include headaches, upper respiratory infections, diarrhea, nasopharyngeal inflammation, elevation in low-density lipoprotein and cholesterol levels and reduction in neutrophil numbers. Serious infections including pneumonia, cellulitis and urinary tract infections have been described in patients treated with Tofacitinib. Of note, Tofacitinib is under additional monitoring for use in the EU by European Medicines Agency due to its association with blood clotting and high rate of infections. In patients in a long-term Tofacitinib clinical trial, the rate of lymphomas and other lymphoproliferative disorders was observed to be 0.07 per 100 patients over the course of each year [175]. There have been 201 clinical trials involving Tofacitinib, of which 16 are in phase IV. Interestingly, there are four clinical trials investigating the potential use of Tofacitinib in COVID-19, which are in the pre-recruiting stage (NCT 04412252, NCT 04415151, NCT 04390061 and NCT 04332042), as well as two trials for its potential therapeutic value in lymphomas and solid tumors (NCT 03598959 and NCT 04034238).

### 3.9. WP1066/WP 1220 (Moleculin Biotech) 

WP1066 is a potent Jak2 inhibitor that suppresses the phosphorylation of Jak2, Stat3, Stat5, as well as ERK1and ERK2, at concentrations of 0.5, 1.0, 2.0, 3.0 and 4.0 μM, without affecting phosphorylation of Jak1 and Jak3 in a cell-free kinase assay (Table 1) [176]. In a cell-based assay, WP1066 inhibited the growth of erythroleukemia HEL cells carrying the mutant Jak2V617F isoform, with the IC50 values of 2.3 μM. Tsujita et al. showed that WP1066 suppressed Stat3 phosphorylation in bladder cancer cell lines and blocked the cell growth of those cells [177]. WP1066 is an analog of a less potent Jak2 inhibitor, AG490, which has been previously shown to inhibit the growth of various cancer cells, such as renal cell carcinoma and leukemia [178,179,180]. WP1066 currently has an orphan drug designation by the FDA for the treatment of brain tumors [181]. Currently, there are two documented clinical trials evaluating the efficacy of WP1066 in patients with brain tumors that are not responsive to current standards of treatment (NCT 01904123, NCT 04334863).

### 3.10. Atiprimod (SKF 106615; AnorMed Inc.)

Atiprimod is an oral small molecule belonging to the Azaspirane family. It has been shown to effectively suppress myeloma cell growth via Stat3 and Jak2 inhibition (Table 1) [182], and it has potential as a treatment for RA and other autoimmune diseases [183]. The inhibition of Jak2 kinase activity by Atiprimod resulted not only in antiproliferative and proapoptotic effects on Jak2V617F-expressing cell lines, such as FDCP-EpoR (murine) and SET2 (human), but also on human megakaryoblastic (CMK) cells carrying the mutation Jak3 A572V within the pseudokinase domain of Jak3. In cell-based assays, the antiproliferative activity of Atiprimod has been shown to be more potent in FDCP cells carrying mutant Jak2V617F (IC50 0.42 µM) and SET-2 cells (IC50 0.53 µM) than in CMK cells carrying mutant Jak3 (IC50 0.79 µM) or FDCP-EpoR WT-Jak2 cells (IC50 0.69 µM) [184]. Of note, the IC50 of Atiprimod was lower in FDCP cells carrying the Jak2V617F mutation (IC50 0.69 µM) than CMK cells with WT-Jak2 [185]. Based on the testing of Atiprimod in cell-based assays, it was also noted that Atiprimod blocked cell growth and induced apoptosis of mantle cell lymphoma cells, while it inhibited in vivo tumor growth and increased survival of mice with mantle cell lymphoma tumors. Moreover, Atiprimod has been shown to have antiproliferative and antiangiogenic activities and induce apoptosis via activation of Caspase-3 and -8 in multiple myeloma (MM) [186]. Furthermore, Atiprimod blocked IL-6-Stat3 pathway in myeloma cells and down-regulated cell proliferation and survival by decreasing levels of the antiapoptotic proteins Bcl-2, Bcl-XL and Mcl-1 [187]. There have been two phase-II clinical trials in patients with advanced carcinoid tumors, one phase-I/IIa clinical trial in relapsed or refractory multiple myeloma sponsored by Callisto Pharmaceuticals (NCT 00388063, NCT 00663429, NCT 00086216), and one phase-I clinical trial for patients with advanced multiple myeloma who had growing tumors and symptoms that were no longer controlled by the standard therapies utilized (NCT 00430014). Interim data from this phase I trial revealed that three of the five patients treated with Atiprimod had significant clinical improvement with a decrease in debilitating symptoms and an increase in tumor regression [186]. Data from a phase I trial of oral Atiprimod in 14 patients with advanced cancers (3 + 3 design) for 14 days every 28-day cycle demonstrated that the most common AEs were related to the gastrointestinal system. Less common AEs were sinus headaches and elevated serum transaminases, alkaline phosphatase and creatinine. 

### 3.11. Ilginatib (NS-018; NS Pharma)

Ilginatib is an oral, selective Jak2 inhibitor with an IC50 of 0.72 nM in a cell-free kinase assay (Table 1). Ilginatib induced inhibition of Stat5 phosphorylation in Mac1^+^/Gr1^+^ myeloid cells from bone marrow samples from Jak2V617F transgenic mice. In an MPN murine model induced by mutated Jak2V617F, hepatosplenomegaly was improved, cell invasion decreased and improved overall survival was observed [188]. An X-ray co-crystal structure revealed that the Ilginatib binds to Jak2 in its active conformation, suggesting that hyper-activated Jak2 is potentially inhibited more effectively than WT-Jak2 [189,190]. Ilginatib is currently being tested in a clinical trial (NCT 01423851) in patients with MF, PCV and post-essential thrombocythemia myelofibrosis-(PET-MF). 

### 3.12. AC430/AC410 (Ambit Biosciences) 

AC430 is an oral, potent Jak2-selective inhibitor intended for autoimmune diseases and cancer with an IC50 value of 28 nM (Table 1) [191]. In preclinical studies, AC430 exhibited potency against Jak2 and Jak2V617F mutation in cell-based models [191]. In preclinical oncology and autoimmune models, AC430 is well tolerated and has significant efficacy at oral doses as low as 10 mg/kg/day [191]. A phase I clinical trial (NCT 01287858) for AC430 on dosing and safety was completed in June 2011 and indicated no serious AEs, with the most common AEs being dysgeusia, GI-related events, fatigue and headache. Importantly, no changes in blood cell counts were observed [191]. There are currently no ongoing clinical trials listed for AC410 [191].

### 3.13. LS104 (AEG 41174; Aegara Bio-Therapeutics) 

LS104 is a novel non-ATP-competitive Jak2 inhibitor developed for the treatment of leukemia and MPD (Table 1) [192]. In a cell-free kinase assay, the IC50 value of LS104 was estimated to be 1500 nM [192]. LS104 treatment of Jak2V617F positive murine hematopoietic Ba/F3 cells results in apoptosis induction in a dose-dependent manner and the inhibition of Jak2 autophosphorylation as well as the inhibition of Jak2 downstream targets [192]. Furthermore, LS104 hinders cytokine-independent growth of primary cells obtained from MPD patients with Jak2V617F mutation in an in vitro endogenous erythroid colonies assay (EEC) that focuses on the terminal erythroid differentiation. LS104 treatment in the murine model of human AML prolonged survival of mice [192]. Based on these positive findings, LS104 received investigative new drug (IND) status from the US FDA leading to a phase I clinical trial in patients with Jak2V617F-positive MPDs [192]. 

### 3.14. Jaktinib (Suzhou Zelgen Biopharmaceuticals) 

Jaktinib is an oral and potent Jak1/2 inhibitor with an IC50 of 0.1 μM based on a cell-free kinase assay (Table 1). It displays similar potency to Momelotinib and Ruxolitinib [193]. Jaktinib suppresses Jakl, Jak2, Jak3 and Tyk2 at the cellular level, inhibits the Jak-Stat signaling pathways to block the release of cytokines, including IL-2, IL-4, IL-6, IL-7 and IL-10, and significantly relieves inflammation due to immune reactions [193]. Preclinical toxicology studies conducted in Wistar rats and Beagle dogs with oral administration of Jaktinib showed no observed AEs [193]. Currently, Jaktinib is in phase II clinical trials for idiopathic pulmonary fibrosis (NCT 04312594), primary MF (NCT 04217993, NCT 03886415) and alopecia areata (NCT 04435392, NCT 04445363). A recent study indicated that Jaktinib has the potential to inhibit the proliferation of SARS-CoV-2 in vivo by inhibiting adapter associated kinase-1 (AAK1) activity, virus replication and cytokine storms to potentially delay disease progression, thereby reducing the mortality of COVID-19 [193]. 

### 3.15. AT9283 (Astex Pharmaceuticals)

AT9283 is a multikinase inhibitor with IC50 in cell-free assays against Aurora Kinase A/B of approximately 3 nM, and the IC50 for Jak2 and Jak3 were 1.2 nM and 1.1 nM, respectively (Table 1, Supplementary Table S1) [179]. Qi et al. demonstrated that AT9283 induced apoptosis and inhibited cell proliferation in B-Non-Hodgkin lymphoma (B-NHL) cell lines at an IC50 < 1 μM in a dose- and time-dependent manner. The same study showed that AT9283 in combination with Docetaxel induced significant apoptosis at a very low dose (5 nM) compared to a single treatment of the B-NHL cells with either of the compounds. In vivo mouse xenograft tumors of mantle cell lymphoma treated with AT9283 (15 or 20 mg/kg) plus Docetaxel (10 mg/kg) showed a significant decrease in tumor growth and enhanced survival of the mice [180]. Similarly, a study in multiple myeloma (MM) demonstrated that a combination of AT9283 with Lenalidomide (orally active immunomodulator) led to significant synergistic cytotoxicity in preclinical models, and the proposed mechanism of action was blocking Stat3 phosphorylation [194]. AT9283 has been tested in five clinical trials, two of which have investigated the potential therapeutic value of AT9283 in solid tumors (NCT 00443976, NCT 00985868). A phase I trial in pediatric patients with solid tumors (unspecified relapsed and refractory solid tumors) revealed that AT9283 was well-tolerated with manageable hematological AEs. The overall notable AEs were GI-side effects (75%), including nausea, diarrhea, constipation, stomatitis and vomiting (19%). Other AEs included pneumonia, fatigue, febrile neutropenia, peripheral edema, headache and epistaxis [195]. AT9283 has been developed by Astex, and it has progressed to a completed phase II trial investigating its potential use in multiple myeloma (NCT 01145989). However, the trial results indicated that AT9283 at a dose of 40 mg/m^2^ and on a schedule (days 1 and 8 of a 21-day cycle) was not suggested for further studies as a treatment for myeloma [195].

### 3.16. Cerdulatinib (PRT062070; Portola Pharmaceuticals)

Cerdulatinib is an orally active, small-molecule inhibitor with activity against 24 kinases with an IC50 range below 200 nM (Table 1, Supplementary Table S1). In a cell-free kinase assay, the IC50 values for Jak1, Jak2, Jak3 and Tyk2 were 12 nM, 6 nM, 8 nM and 0.5 nM, respectively. Further studies showed that Cerdulatinib induced significant apoptosis in NHL-cell lines expressing B-cell antigen receptor (BCR)-signaling. Furthermore, Cerdulatinib significantly inhibited the viability of CD19^+^ cells isolated from 60 primary chronic lymphocytic leukemia (CLL) patient samples at clinically attainable concentrations. In those 60 primary CLL samples, the IC50 of Cerdulatinib ranged from 0.4 to 10.0 µM with cell viability as the endpoint, and the average IC50 for the cohort was 2.6 μM and the median IC50 was 1.5 μM. According to a clinical pharmacokinetic study, these concentrations are clinically achievable [196]. Additionally, Ibrutinib-resistant primary CLL cell growth was inhibited by Cerdulatinib, and these anti-tumor effects were proposed to be linked to inhibition of BCR, IL-4/Jak1/Jak3/Stat6 and IL-6/Jak1/Jak2/Stat3 signaling [196]. Cerdulatinib has progressed into a phase IIb clinical trial studying relapsed/refractory peripheral T-cell lymphoma (PTCL) (NCT 04021082), a phase IIa clinical trial to assess the safety and tolerability for patients with vitiligo (NCT 04103060); and a phase I/IIa dose-escalation study in CLL, small lymphocytic lymphoma (SLL) or B-cell non-Hodgkin lymphoma (NHL) (NCT 01994382). A next clinical trial (NCT 04757259) is currently available for eligible patients with relapsed/refractory CLL or NHL for patients who experienced clinical benefit from Cerdulatinib (NCT 01994382). 

### 3.17. Filgotinib (GLPG0634/GS-6034/Jyseleca; Galapagos NV)

Filgotinib is a selective Jak inhibitor that demonstrates potent activity for Jak1 over Jak2, Jak3 and Tyk2 with IC50 values of 10 nM, 28 nM, 810 nM and 116 nM, respectively (Table 1, Supplementary Table S1) [147]. Further, cell-based assays revealed that Filgotinib and its active metabolites block Jak1-dependent cytokine signaling pathways [197]. In a preclinical setting, Filgotinib inhibited Stat3 and the expression of the oncostatin-M receptor (OSMR) in oncogene-driven NSCLC [198]. Filgotinib is currently being developed for the treatment of RA, psoriatic arthritis, ankylosing spondylitis and inflammatory bowel disease. Filgotinib has progressed into several phase II and phase III studies, mostly investigating its potential in RA [199]. Filgotinib received its first approval in the EU [200] and Japan [201] in 2020 for the treatment of moderate to severe active RA in adults. However, pre-clinical animal studies showed that Filgotinib impaired spermatogenesis. Therefore, US FDA has requested data from two ongoing studies, the MANTA (NCT 03201445) and MANTA-Ray (NCT 03926195), to assess whether Filgotinib has an impact on sperm parameters in adult males [202]. Among 448 patients treated with Filgotinib, 1% developed herpes zoster retinal vein occlusion. Common side effects noted so far include nasopharyngitis (10.2%) and headache and nasopharyngitis (5.9% each) [203]. 

### 3.18. Decernotinib (VX 509; Vertex Pharmaceuticals) 

Decernotinib is an oral selective Jak3 inhibitor developed for the treatment of RA (Table 1, Supplementary Table S1) [204]. Animal pharmacology studies in a rat collagen-induced arthritis model (CIA) demonstrated that inflammation was significantly reduced in a dose-dependent manner. Many of the physiological mechanisms associated with RA, such as bone resorption and cartilage damage, were also reduced [204]. Decernotinib was evaluated in six phase-II clinical trials (NCT 01830985, NCT 01886209, NCT 01754935, NCT 01590459, NCT 00789126, NCT 01052194). Decernotinib has been shown to be well tolerated except for increased opportunistic infections, elevation in the levels of transaminase and creatinine, as well as lipoproteins in blood [205]. Vertex has discontinued the clinical development of Decernotinib for RA [205]. 

### 3.19. Erlotinib (Tarceva; Genentech)

Erlotinib is an oral EGFR (tyrosine kinase) inhibitor with potent off-target Jak2 inhibitory effects (Table 1, Supplementary Table S1). Erlotinib was initially developed for therapy in patients with NSCLC, as it has been documented to inhibit the growth of Jak2V617F positive hematopoietic progenitor cells and human erythroleukemia (HEL cells). In a study investigating the potency of Erlotinib in PCV in a cell-based assay, WT-Jak2 was inhibited with an IC50 of >20 μM and Jak2V617F at 4 μM [206]. Of particular interest, Erlotinib has been investigated clinically in PC with an emphasis on EGFR as a primary target. Overall, in a phase II study of 30 patients with PC (29 with CRPC, 23 of which had been treated with prior chemotherapy), a clinical benefit was shown in 40% of patients with an endpoint of a decrease in PSA without clinical progression. Another similar study enrolled 29 patients and showed that there was a moderate clinical improvement in chemotherapy naïve CRPC defined by a decrease in PSA levels [207]. There are 589 clinical trials for Erlotinib investigating its use in many various cancers, 74 of which pertain to solid tumors, 8 for PC. Erlotinib has been FDA approved for over a decade in NSCLC and is produced by Genentech. Most common AEs related to Erlotinib included cutaneous changes such as rash and hair loss, as well as other neurogenic inflammation [208].

### 3.20. Givinostat (ITF2357; ItalFarmaco) 

Givinostat is primarily an oral, histone-deacetylase (HDAC) inhibitor as well as a potent inhibitor of hematopoietic colony formation by Jak2V617F-expressing progenitor cells from chronic MPN in vitro (Table 1, Supplementary Table S1). Givinostat was well tolerated in MF patients in a phase II trial. Side effects observed were GI-related and fatigue [209]. Givinostat treatment resulted in neoplastic inhibition in MF and MPNs. Proliferation was inhibited and apoptosis was induced in vitro in B-cell acute lymphoblastic leukemia (BCP-ALL) cytokine receptor-like factor 2 (CRLF2) cell lines without the induction of death of normal hematopoietic cells. Givinostat has been reported to downregulate Jak-Stat pathways leading to a significant decrease in Stat5 phosphorylation. In vivo, Givinostat significantly reduced engraftment of human blasts in patient-derived xenograft models of CRLF2-positive BCP-ALL cells. Additionally, Givinostat killed Ruxolitinib-resistant cells and potentiated the effect of current chemotherapy [210]. Givinostat is being tested in 17 ongoing clinical trials, mainly in patients with PCV, muscular dystrophy, MPNs, lymphomas or arthritis. Three phase-II clinical trials have been completed for Givinostat: Two for PCV (NCT 01901432, NCT 00928707) and one for Duchenne muscular dystrophy (DMD) (NCT 01761292). One phase-II clinical trial for polyarticular course juvenile idiopathic arthritis (NCT 01261624) has been terminated. 

### 3.21. Repotrectinib (TPX0005; Turning Point Therapeutics)

Repotrectinib is a potent inhibitor of Ros1, Trk and Alk (Table 1, Supplementary Table S1). Other clinically relevant kinases inhibited by Repotrectinib are Jak2, Lyn, Src and Fak. The IC50 in a cell-free assay of Repotrectinib against Ros1, TrkA, TrkB, TrkC, Alk, Jak2, Lyn, Src and Fak were 0.07 nM, 0.83 nM, 0.05 nM, 0.1 nM, 1.01 nM, 1.04 nM, 1.66 nM, 5.3 nM and 6.96 nM, respectively [211,212]. The off-target effects of Repotrectinib on Jak2 may potentially provide a longer duration of action and inhibit the development of resistance mechanisms in NSCLC. Repotrectinib inhibited proliferation of Ba/F3 cells in vitro, and tumor volumes were decreased in mice expressing Ba/F3 Cd74–Ros1-WT or Cd74-Ros1-G2032R xenograft tumors. In addition, Repotrectinib inhibited Stat5 phosphorylation with IC50 of 158 nM in SET2 cells and Src phosphorylation with IC50 of 89 nM in H2228 cells [213]. There have been three clinical trials for Repotrectinib: One in phase I for NSCLC (NCT 04772235) and two clinical trials in phase II for pediatric patients harboring Alk, Ros1 or Ntrk1-3 mutations in solid tumors, lymphomas and central nervous system tumors (NCT 04094610), and adult patients with advanced solid malignancies harboring an Alk, Ros1, Ntrk1, Ntrk2 or Ntrk3 gene rearrangement (NCT 03093116). In the currently available data, Repotrectinib was generally well tolerated by oral administration in patients.

### 3.22. Zotiraciclib (TG02; Adastra Pharmaceuticals)

Zotiraciclib is a pyrimidine-based multi-kinase inhibitor that inhibits CDK 1, CDK2, CDK7 and CDK9 as well as Jak2 and FLT3 (Table 1, Supplementary Table S1) [214]. The primary molecular targets of Zotiraciclib are CDK proteins, but Zotiraciclib has also been shown to inhibit Jak2 and FLT3 in cancer cells [214]. Zotiraciclib has high potency against CDKs with an IC50 of 3–9 nM, and the IC50s for Jak1/Jak2/Tyk2 were between 14 and 59 nM in cell-free assays [215]. In cell-based assays, the growth of patient-derived acute myeloid leukemia (AML) and PCV primary cell lines was effectively inhibited [214]. Studies utilizing AML xenograft models in vivo confirmed the in vitro findings, wherein Zotiraciclib induced effective blockade of Stat3, Stat5 and CDK signaling leading to tumor regression and prolonged survival of mice carrying the tumors [214]. Preclinical studies in glioblastoma cells demonstrated that Zotiraciclib decreased cellular ATP production by suppressing glycolysis, leading to mitochondrial dysfunction resulting in glioblastoma cell death. In December 2019, the FDA granted orphan drug status to Zotiraciclib for use in patients with glioblastoma [214]. This designation was based on the results from the NCI-sponsored phase I/II trial at the NIH Clinical Center (NCT 02942264) [214]. 

## 4. Next-Generation Type I Jak2-Inhibitors Currently in Pre-Clinical Development 

### 4.1. NVP-BSK805 (Novartis) 

NVP-BSK805 is an ATP-competitive Jak2 inhibitor with IC50s of 0.5 nM, 32 nM, 19 nM and 11 nM in a cell-free assay for Jak2, Jak1, Jak3 and Tyk2, respectively (Table 2) [216]. NVP-BSK805 suppressed growth, induced apoptosis and inhibited constitutive Stat5 phosphorylation at IC50 < 100 nM in cells expressing mutated Jak2V617F [216]. In contrast, greater than 1 μM concentrations of NVP-BSK805 were required to inhibit growth and Stat5 phosphorylation in K562 cells carrying a BCR-ABL fusion [216]. In a Ba/F3 Jak2V617F cell-driven mouse model, NVP-BSK805 was efficacious in suppressing Stat5 phosphorylation, leukemic cell spreading and splenomegaly at 150 mg/kg by oral administration. Additionally, NVP-BSK805 suppressed recombinant human erythropoietin-induced polycythemia and extramedullary erythropoiesis in mice and rats at doses of 25, 50 and 100 mg/kg by oral administration [216]. There are no clinical trials listed for NVP-BSK805 at this time.

### 4.2. CEP-33779 (Cephalon Inc./Teva Pharmaceuticals) 

CEP-33779 is a highly selective, orally bioavailable Jak2 inhibitor (Table 2). The IC50 of Jak2 is 1.8 nM, and for other Jak family members, CEP-33779 demonstrated varying levels of selectivity from >40-fold versus Jak1 to >800-fold against Tyk2 in a cell-free assay. In two RA mouse models, Jak2 inhibition by CEP-33779 decreased the mean paw edema and clinical scores [217]. Another in vivo study demonstrated that CEP-33779 treatment (10 mg/kg, 30 mg/kg and 55 mg/kg) resulted in significant inhibition of established colorectal tumors by decreasing angiogenesis and proliferation of tumor cells. Furthermore, the reduced tumor burden was linked to decreased Stat3 and NF-κB (RelA/p65) activation [218]. Although preclinical results have been promising, there are currently no listed clinical trials.

### 4.3. TG101209 (TargeGen/Sanofi)

TG101209 is a selective small-molecule inhibitor of Jak2, which potently inhibits mutations associated with MPDs (Table 2). TG101209 inhibits Jak2, FLT3 and RET kinases at IC50 values of 6 nM, 25 nM and 17 nM, respectively. However, TG101209 showed substantially less activity against other tyrosine kinases, including Jak3 IC50 of 169 nM [219]. In vitro studies showed that Ba/F3 cell growth (Jak2V617F) was inhibited at IC50 of 200 nM. Furthermore, TG101209 directly induced cell cycle arrest, apoptosis and blocked the phosphorylation of Jak2V617F, Stat5 and Stat3 in myeloid leukemia cells [219]. In vivo studies demonstrated that SCID mice injected intravenously with Ba/F3 cells expressing Jak2V617F exhibited a significant decrease in Stat5 phosphorylation in splenic tumors after the administration of two oral doses of TG101209 (50 mg/kg) [220]. Another study showed that in lung cancer cell lines HCC2429 and H460, TG101209 inhibited STAT3 activation and surviving expression and sensitized the cells to radiation in clonogenic assays. Furthermore, the addition of TG101209 to radiation delayed the growth of lung cancer xenograft tumors by increasing apoptosis and decreasing cell proliferation [221]. Moreover, T-cell-ALL treated with TG101209 significantly inhibited T-ALL cell proliferation and induced cell apoptosis in a dose-dependent manner [222]. Even though these preclinical data with TG101209 have been highly promising, there are no current clinical trials.

### 4.4. AZ960 (AstraZeneca) 

AZ960 is a highly potent ATP competitive Jak2 inhibitor with IC50 values against Jak2 and Jak3 of 3 nM and 9 nM, respectively, in a cell-free assay (Table 2) [223]. In addition, AZ960 has been shown to have inhibitory activity against other kinases, including Trk-A, Aurora-A and Fak, with IC50 of 100 nM [224]. In cell-based assays, AZD960 inhibited Jak2 and Stat5 phosphorylation and down-regulated proliferation of Ba/F3 cells expressing TEL-Jak2 (catalytic domain of Jak2 fused with the oligomerization domain of the TEL protein, resulting in constitutive activation of the Jak2 kinase activity) [224]. In addition, AZD960 demonstrated selectivity for TEL-Jak2-driven Stat5 phosphorylation and cell proliferation when compared with cell lines driven by similar fusions of the other Jak kinases (Jak1, Jak3 and Tyk2) [224]. Moreover, AZD960 suppressed constitutive and inducible Stat3 activation and as well as the growth of xenograft tumors of breast, ovarian and PC origin in mice, suggesting that Jak2 is required for Stat3 activation in solid tumors [225]. Moreover, AZD960 suppressed constitutive and inducible Stat3 activation and as well as the growth of xenograft tumors of breast, ovarian and PC origin in mice, suggesting that Jak2 is required for Stat3 activation in solid tumors [223]. There are no clinical trials testing the safety or efficacy of AZ960 in any tumor type.

### 4.5. CHZ868 (Novartis)

Novartis launched a discovery program to identify type II Jak2 inhibitors with improved potency, selectivity and physicochemical properties, which led to the discovery of CHZ868 (Table 2) [226]. CHZ868 is a Type II Jak2 inhibitor that stabilizes Jak2 in an inactive conformation and prevents Jak2 autophosphorylation [227]. In a comparison between Jak2V617F expressing SET-2 cells and mutant Jak3A572V expressing acute myeloblastic leukemia (MKB) cells, CHZ868 was highly selective for Jak2 over other Jak family members [227]. In vivo, CHZ868 demonstrated favorable efficacy in reducing growth in the Jak2V617F knock-in mouse model of PCV and, similarly, in MF mouse models [227]. A key finding related to CHZ868 was that withdrawal of CHZ868 did not trigger Stat rebound signaling as has been observed for Type I Jak2 inhibitors [227]. The same study showed that CHZ868 potently suppressed the growth of MHH-CALL4 leukemia cells with IC50 values in the range of 100 nM, while 1 μM of CHZ868 suppressed Jak2 phosphorylation, suggesting the involvement of other signaling pathways than Jak2 in the growth regulation of these cells [227]. There are no clinical trials ongoing for CHZ868 due to toxicity in preclinical models.

### 4.6. ON044580 (Onconova Therapeutics)

ON044580 is a non-ATP mimetic (Type I) Jak2 and BCR-ABL kinases inhibitor (Table 2). Studies in vitro, using a recombinant Jak2 protein, showed that ON044580 inhibited the kinase activity of recombinant Jak2 with an IC50 between 0.9 and 1.2 µM in a cell-free assay [228]. ON044580 was also able to inhibit the activated Jak2V617F mutant with an IC50 between 0.8–1.1 µM [228]. In the same assay, it was shown that the JH2 domain is necessary for ON044580 to effectively inhibit Jak2 [228]. In cell-based assays, the suppression of Jak2 and Stat5 phosphorylation was documented within 30 min of compound administration to Jak2V617F-positive leukemia cells. This caused both growth arrest and apoptosis within human and mouse leukemia cells positive for Jak2V617F [228]. Patient-derived CML cells that were refractory to Imatinib (BCR-ABL kinase inhibitor) displayed high sensitivity to ON044580. In BCR-ABL+ cells, ON044580 induced apoptosis led to a rapid decrease in BCR-ABL protein levels [229]. Consequently, targeting Jak2 and BCR-ABL kinases with ON044580 has the potential to manage multiple types of drug-resistant CML cells where tyrosine kinase inhibitors are not clinically useful. There are no clinical trials reported at this time [229]. 

### 4.7. ZT55 (Molnova)

ZT55 is a highly selective Jak2 inhibitor with IC50 of 0.031 μM against Jak2 in a cell-free kinase assay (Table 2) [230]. ZT55 exhibits potent effects on the Jak-Stat pathway, downstream Stat3/5 transcription factors and inhibits tyrosine phosphorylation in Jak2V617F mutation on HEL cell line [230]. In addition, ZT55 effectively inhibited proliferation of HEL cell line expressing Jak2V617F mutation, followed by cell cycle arrest at G2/M phase leading to caspase-dependent apoptosis [230]. The growth of HEL xenograft tumors in vivo was significantly suppressed by ZT55 treatment [230]. Further evaluation indicated inhibition of erythroid colony formation of peripheral blood hematopoietic progenitor cells from patients carrying the Jak2V617F mutation by ZT55 [230]. No current clinical trials have been listed. 

## 5. Next-Generation Type I Jak2-Inhibitors with the Clinical Development Terminated 

### 5.1. AZD1480 (AstraZeneca)

AZD1480 is a potent, ATP mimetic inhibitor of Jak2 with an IC50 of less than 0.4 nM in a cell-free assay (Table 3). In cell-based assays and in various xenograft tumor models, AZD1480 demonstrated inhibition of Stat3 and Stat5 [231]. In particular, AZD1480 potently inhibited both Stat5 and Stat3 in preclinical models of PC, resulting in reduced PC cell viability and CRPC tumor growth after androgen deprivation conducted by surgical castration [47,84,232]. Higher concentrations of AZD1480 have been shown to suppress Aurora A/B Kinase and histone H3 phosphorylation [233]. In addition, the proliferation of myeloma cells was potently inhibited in vitro and in vivo secondary to the inhibition of phosphorylated FGFR3, phosphorylation of Jak2 and Stat3 and Cyclin D2 by AZD1480 [233]. AZD1480 was tested in three clinical trials investigating its potential safety and efficacy in treating solid tumors (NCT 01219543, NCT 01112397) and MF/PCV (NCT 00910728). The preliminary results led to early termination due to reversible neurotoxicity [233]. 

### 5.2. XL019 (Exelixis Inc.) 

XL019 is a potent, orally active and selective Jak2 inhibitor, with IC50 of 2.2 nM, 134.3 nM and 214.2 nM for Jak2, Jak1 and Jak3, respectively, based on a cell-free kinase assay (Table 3) [234]. XL019 inhibits Stat3 and Stat5 phosphorylation in cells expressing either Jak2V617F or WT-Jak2 [234]. In addition, XL109 demonstrated efficacy in AML xenograft tumor mouse models [235]. XL019 was evaluated in two phase-I clinical trials in patients with PCV or MF (NCT 00595829 and NCT 00522574), which were both terminated due to safety concerns related to neurotoxicity in the patient [235]. 

### 5.3. BMS-911543 (Bristol-Myers Squibb) 

BMS-911543 is a highly selective small-molecule inhibitor of Jak2 with 74-fold selectivity over Jak3 and 350-fold selectively over Jak1 (Table 3). In a cell-free assay, the IC50 values of BMS-911543 for Jak1, Jak2, Jak3 and Tyk2 were 356 nM, 1.1 nM, 73 nM and 66 nM, respectively [236]. BMS-911543 has been shown to induce anti-proliferative responses in SET-2 and BaF3 cell lines, both engineered to express Jak2V617F, compared to non-Jak2-dependent cell lines (A549, MDA-MB-231, MiaPaCa-2) [237]. Furthermore, BMS-911543 blocked the growth of primary progenitor cells isolated from patients with Jak2V617F-positive MPNs in a colony formation assay [238]. BMS-911543 has been tested in one clinical trial (NCT 01236352), which was terminated.

### 5.4. Tozasertib (MK-0457/VX680; Vertex Pharmaceuticals/Merck) 

Tozasertib is the first potent Aurora kinase inhibitor that has been tested in clinical trials (Table 3). Preclinical studies demonstrated that Tozasertib inhibited both Aurora kinase A and B activity [239,240,241]. Other kinases that have been described as molecular targets of Tozasertib are FLT-3 and ABL and Jak2 kinase [239]. Upon treatment with Tozasertib, inhibition of cell growth and increased apoptosis have been reported in PC and thyroid, ovarian and oral squamous cancer cell lines [240,241,242]. Tozasertib also reduces tumor growth in an in vivo model of PC [241]. Tozasertib has been evaluated in seven clinical trials for efficacy in hematological malignancies, advanced solid tumors, colorectal cancer and NSCLC. Although the preliminary clinical results were promising, the clinical development has been terminated with only one completed trial in patients with leukemia (NCT 00111683) with concerns for neutropenia, thrombocytopenia and anemia [239,241].

## 6. Conclusions

Most of the Jak2 inhibitors show high efficacy against WT-Jak2 in cell-free assays but lack evidence for efficacy against WT-Jak2 in cell-based assays. In other words, the cell-based assays that have been utilized to test the efficacy/potency of Jak2 inhibitors are cell/tumor models expressing mutated Jak2V617F. In this regard, it is important to note that the mutated Jak2V617F is not expressed in clinical PC. The Jak2 inhibitors with higher potency or exclusive potency against mutated Jak2V617F compared to WT-Jak2 are not likely to have significant efficacy against PC growth. The third key point is that most Jak2-inhibitors in preclinical and clinical development do have simultaneous inhibitory activity against Jak1. However, since Stat3 is pro-oncogenic in PC cells, the simultaneous inhibition of Jak1/Jak2-Stat3 axis is actually beneficial for the desired outcome of blocking PC growth and potential metastatic progression.

In terms of AEs, most of the next-generation Jak2 inhibitors are relatively well tolerated with the AEs, mainly including GI effects, fatigue, opportunistic infections and anemia. The clinical development of many of the first-generation Jak2 inhibitors has been discontinued because of neurotoxicity. Overall, there is substantial evidence to support chemotherapeutic use of Jak2/Stat5 inhibitors for CRPC from both preclinical and clinical studies. Many of the next-generation Type I Jak2 inhibitors are well tolerated with oral bioavailability. In summary, evaluation in phase I/II clinical trials is warranted to determine the potential efficacy of the next-generation Type I Jak2 inhibitors that have demonstrated efficacy in cell-based assays against WT-Jak2 in PC.

In summary, the key challenges related to the first-generation Jak2 inhibitors included neurotoxicity due to the ability of those compounds to cross the blood–brain barrier. This was followed by the development of a set of next-generation Type I Jak2 inhibitors with decreased neurotoxicity and improved safety profiles. However, many of the next-generation Jak1/2 inhibitors in the clinical development are associated with increased infection rates and yet unknown effects on the host immune system and, for example, the gut microbiota that warrant additional studies. Both Fedratinib and Pacritinib are furthest along as Type I next-generation Jak2 inhibitors for oral administration in the clinical development and with largely manageable AE profiles.

## Figures and Tables

**Table 1:** JAK Family Inhibitors in Clinical Development.

Drug Name	Mechanism	Indication	NCT #	Trial Phase	Outcome
Ruxolitinib * (FDA approved)	Jak1 > Jak2 > Tyk2 > Jak3 inhibitor (Type 1) [105]	Prostate cancer	NCT 03274778	N/A	Withdrawn: Recruitment difficulty
		Refractory malignant solid neoplasm	NCT 03878524	1	Recruiting
		Androgen-independent prostate cancer	NCT 00638378	2	Terminated: Low efficacy
		Prostate cancer	NCT 03274778	N/A	Withdrawn: Recruitment difficulty
		Solid tumors (including prostate)	NCT 02711137	1/2	Terminated
Fedratinib * (FDA approved)	Jak2 inhibitor (Type 1) [106]	Solid tumors	NCT 01836705	1	Completed
		Solid tumors	NCT 01585623	1	Completed
Pacritinib *	Jak2 (Type 1) [107]	MF, PCV, post-essential thrombocythemia MF	NCT 02055781	3	Terminated (FDA concerns)
		MF, PCV, post-essential thrombocythemia MF	NCT 03165734	3	Recruiting
		COVID	NCT 04404361	3	Recruiting
		MF, PCV, post-essential thrombocythemia MF	NCT 02055781	3	Terminated (FDA concerns)
		MF, PCV, post-essential thrombocythemia MF	NCT 01773187	3	Terminated (FDA concerns)
		NSCLC	NCT 02342353	1	Terminated (drug shortage)
		Refractory colorectal cancer	NCT 02277093	2	Terminated (increased side effects)
Baricitinib (FDA approved)	Jak1, Jak2 inhibitor (Type 1)	RA	NCT 01711359	3	Completed
		RA	NCT 01721057	3	Completed
		COVID-19	NCT 04358614	2/3	Completed
		RA	NCT 01710358	3	Completed
Momelotinib *	Jak1, Jak2 inhibitor (Type 1) [108]	Symptomatic anemic MF	NCT 04173494	3	Recruiting
		Thrombocytopenia and MF	NCT 02101268	3	Completed
		MF	NCT 01969838	3	Completed
		Untreated metastatic pancreatic ductal adenocarcinoma	NCT 02101021	3	Terminated (sponsor withdrew)
		Adjuvant capecitabine and oxaliplatin in pancreatic ductal adenocarcinoma	NCT 02244489	1	Terminated
		NSCLC	NCT 02206763	1	Terminated
		Safety/Efficacy in PCV	NCT 01998828	2	Terminated
		NSCLC	NCT 02258607	1	Terminated
Gandotinib	Jak2 inhibitor (Type 1)	MF, PCV, ET	NCT 01594723	2	Active
		MF, PCV, ET	NCT 01520220	1	Completed
		Healthy males	NCT 01577355	1	Completed
		MF, PCV, ET	NCT 01134120	1	Completed
Peficitinib (FDA approved)	Pan-Jak inhibitor (Type 1)	RA	NCT 01638013	3	Completed
		RA	NCT 02308163	3	Completed
		RA	NCT 02305849	3	Completed
Lestaurtinib *	FTL3 inhibitor (Jak2 off target inhibitor)	Acute lymphoblastic leukemia	NCT 00557193	3	Active, not yet recruiting
		Asymptomatic hormone-refractory prostate cancer	NCT 00081601	2	Completed
		Acute lymphoblastic leukemia	NCT 00557193	3	Active, not yet recruiting
		High risk neuroblastoma	NCT 00084422	1	Completed
Tofacitinib * (FDA approved)	Jak3 > Jak2 > Jak1 inhibitor (Type 1) [109]	Previously treated pancreatic adenocarcinoma, cholangiocarcinoma and other mesothelin expressing solid tumors	NCT 04034238	1	Recruiting
	Jak3 > Jak2 > Jak1 inhibitor (Type 1) [109]	Relapsed and refractory extranodal NK/T-cell lymphoma	NCT 03598959	2	Not yet recruiting
WP 1066 *	Jak2 inhibitor	Recurrent/progressive pediatric brain tumor	NCT 04334863	1	Recruiting
		Recurrent malignant glioma or Progressive metastatic brain melanoma (18+)	NCT 01904123	1	Recruiting
Atiprimod *	Jak2, Jak3 inhibitor	Neuroendocrine carcinoma	NCT 00388063	2	Completed
		Neuroendocrine carcinoma	NCT 00663429	2	Completed
		Multiple myeloma	NCT 00086216	1/2	Completed
		Advanced cancer	NCT 00430014	1	Terminated: Sponsor withdrew
		Advanced cancer	NCT 00214838	1/2	Unknown, not recruiting.
Ilginatib (NS-018)	Jak2 inhibitor (Type 1)	MF, PCV and post-ET MF	NCT 01423851	1/2	Active
AC430	Jak2 inhibitor	Safety in healthy subjects	NCT 01287858	1	Completed
LS104	Jak2 inhibitor (allosteric)	Hematological malignancies	Unavailable	1	Unknown
		Hematological malignancies	Unavailable	1	Unknown
Jaktinib	Jak2 inhibitor	Safety trial in healthy volunteers	NCT 03314402	1	Completed
		MF post Ruxolitinib Intolerance	NCT 04217993	2	Recruiting
		Intermediate and high-risk MF	NCT 03886415	2	Recruiting
AT9283 *	Aurora kinase inhibitor (Jak2 off target)	Non-Hodgkin’s lymphoma and solid tumors	NCT 00443976	1	Completed
		Relapsed or refractory multiple myeloma	NCT 01145989	2	Completed
		Relapsed or refractory acute leukemia	NCT 01431664	1	Completed
		Relapsed or refractory solid tumors in pediatric patients	NCT 00985868	1	Completed
		Leukemia dose escalation	NCT 00522990	1/2	Terminated (phase II dose determined)
Cerdulatinib *	SYK and JAK inhibitor	Vitiligo	NCT 04103060	2	Recruiting
		Chronic lymphocytic leukemia, Non-Hodgkin lymphoma	NCT 01994382	1/2	Recruiting
		Peripheral T-cell lymphoma	NCT 04021082	2/3	Withdrawn by sponsor, not initiated
Filgotinib (FDA approved)	Jak1 inhibitor (Type 1)	Ulcerative colitis	NCT 02914522	3	Completed
		RA	NCT 02873936	3	Completed
		RA	NCT 02886728	3	Completed
		RA	NCT 02889796	3	Completed
		Testicular safety	NCT 03201445	2	Recruiting
Decernotinib	Jak3 inhibitor (Type 1)	RA	NCT 01830985	2/3	Completed
	Jak3 inhibitor (Type 1)	Healthy subjects	NCT 01886209	1	Completed
		RA	NCT 01886209	2	Completed
		RA	NCT 01590459	2	Completed
		Healthy subjects	NCT 00789126	1	Completed
		RA	NCT 01052194	2	Completed
Erlotinib *	EGFR inhibitor (Jak2 off target inhibitor)	Chemo-naive, androgen independent prostate cancer	NCT 00272038	2	Completed
		Adjuvant bevacizumab in prostate cancer	NCT 00203424	2	Completed
		Non-metastatic prostate cancer with rising PSA	NCT 00148772	2	Completed
		Adjuvant docetaxel in older patients with prostate cancer	NCT 00087035	2	Completed
		Solid tumors and liver/kidney dysfunction	NCT 00030498	1	Completed
		Dose escalation study	NCT 00739453	1b	Completed
		Drug combination study in various cancers	NCT 03878524	1	
		Adjuvant bevacizumab in hormone refractory prostate cancer	NCT00996502	1/2	Terminated
Givinostat *	HDAC inhibitor	R/R Hodgkin’s lymphoma	NCT 00792467	1/2	Completed
		Jak2 V617F positive chronic myeloproliferative diseases	NCT 00606307	2	Completed
		Chronic myeloproliferative neoplasms	NCT 01761968	2	Active
		R/R Hodgkin’s lymphoma	NCT 00496431	1/2	Terminated: Well-tolerated with low efficacy
Repotrectinib *	ROS1 inhibitor with Jak2 (off target)	Solid tumors	NCT 03093116	1/2	Recruiting
Zotiraciclib *	CDK and Jak1,2 inhibitor	Adults with recurrent anaplastic astrocytoma and glioblastoma	NCT 02942264	1/2	Recruiting

Current state of the clinical development of Jak1 and Jak2 inhibitors. Information in this chart has been sourced from ClinicalTrials.gov. * Indicates documented investigation in solid tumors. Abbreviations: NK: Natural Killer, RA: Rheumatoid Arthritis, UC: Ulcerative Colitis, PC: Prostate Cancer, CRPC: Castrate-Resistant Prostate Cancer. nmPC: Non-metastatic Prostate Cancer, NSCLC: Non-small Cell Lung Carcinoma, MF: Myelofibrosis, PCV: Polycythemia Vera, ET: Essential Thrombocythemia, PSA: Prostate Specific Antigen, R/R: Refractory/Relapsed.

**Table 2:** JAK Family Inhibitors not in Clinical Development.

Drug Name	Mechanism	Indication	NCT #	Trial Phase	Outcome
NVP-BSK805	Jak2 inhibitor (Type 1)			0	
CEP-33779	Jak2 > Jak3 inhibitor (Type 1)			0	
TG101209	Jak2 inhibitor (type I)			0	
AZ960	Jak2 inhibitor (Type 1)			0	
CHZ868	Jak2 inhibitor (Type 2)			0	
ON044580	Jak2/BCR-ABL dual inhibitor (allosteric)			0	
ZT55	Jak2 inhibitor			0	

**Table 3:** Next-Generation Type I Jak2-inhibitors with terminated clinical development.

Drug Name	Mechanism	Indication	NCT #	Trial Phase	Outcome
AZD1480	Jak1/2 inhibitor (Type I) [110]	Primary myelofibrosis and post-PCV/ET-MF	NCT 00910728	1	Terminated (toxicity)
		Solid tumors, gastric cancer, HCC, NSCLC	NCT 01219543	1	Terminated (toxicity)
		Solid tumors	NCT 01112397	1	Terminated (toxicity)
XL019	Jak2 inhibitor (Type 1)	PCV	NCT 00595829	1	Terminated (toxicity)
		MF	NCT 00522574	1	Terminated (toxicity)
BMS-911543	Jak2 inhibitor	MF	NCT 01236352	1/2	Terminated (business decision)
MK-0457/VX680	Aurora kinase inhibitor		NCT 00111683	1	Terminated (toxicity)
			NCT 02532868	1	Terminated (toxicity)
			NCT 00290550	2a	Terminated (toxicity)
			NCT 00405054	2	Terminated (toxicity)
			NCT 00099346	1	Terminated (toxicity)
			NCT 00500006	1	Terminated (toxicity)

Information in this chart has been sourced from ClinicalTrials.gov. Abbreviations: HCC: Hepatocellular carcinoma, NSCLC: Non-small Cell Lung Carcinoma, MF: Myelofibrosis, PCV: Polycythemia Vera, ET: Essential Thrombocythemia.

## Supplementary Materials

The following are available online at https://www.mdpi.com/article/10.3390/cancers13205204/s1, Table S1: Chemical structures of JAK Family Inhibitors in Clinical Development.
