# Supplementary material for: Second-Generation Jak2 Inhibitors for Advanced Prostate Cancer: Are We Ready for Clinical Development?
Source: Cancers (Basel). 2021 Oct 17;13(20):5204. doi: 10.3390/cancers13205204 (PMC8533841; doi:10.3390/cancers13205204)
Supplement: Supplementary file 1 [file cancers-13-05204-s001.zip › cancers-1395550-supplementary.pdf]

Supplementary Table S1. Chemical structures of JAK Family Inhibitors in Clinical Development.

Drug Name	Chemical structure
Ruxolitinib	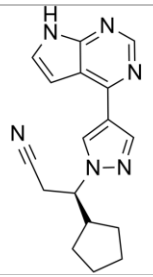
Fedratinib	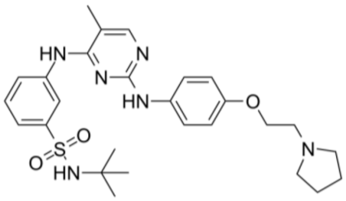
Pacritinib	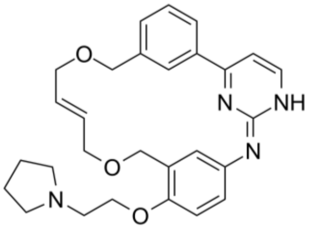
Baricitinib	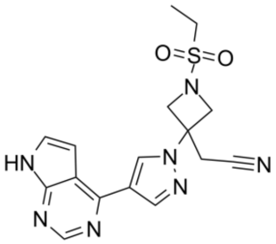
Momelotinib	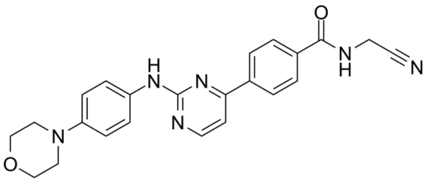

Gandotinib	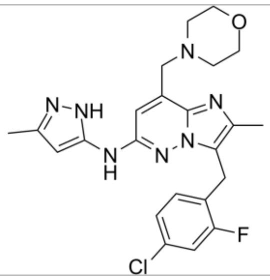
Peficitinib	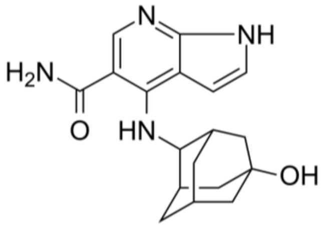
Lestaurtinib	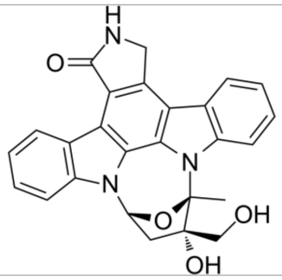
Tofacitinib	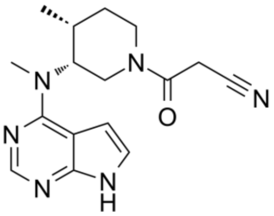
WP 1066	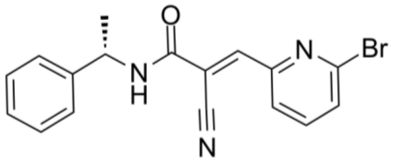
Atiprimod	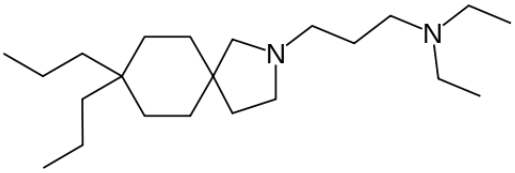

Ilginatib (NS-018)	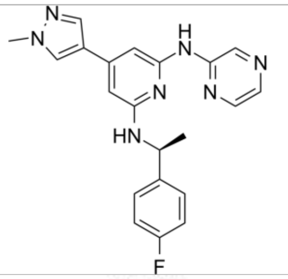
AC430	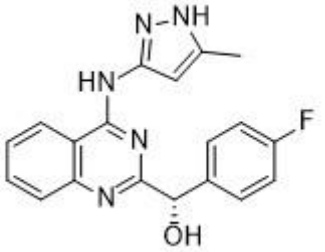
LS104	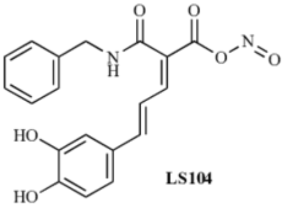
AT9283	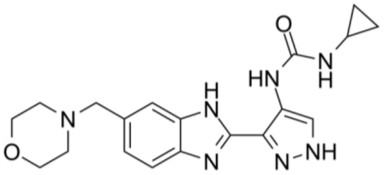
Cerdulatinib	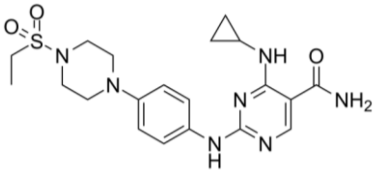
Filgotinib	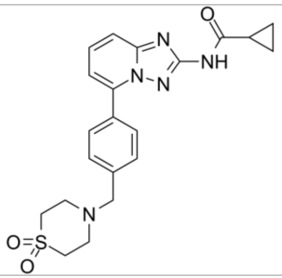
Decernotinib	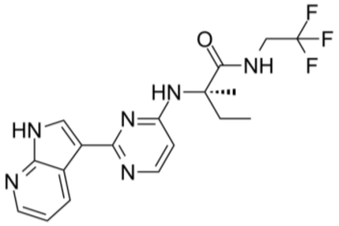

Erlotinib	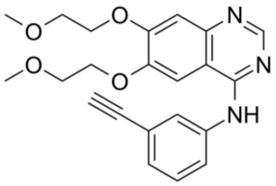
Givinostat	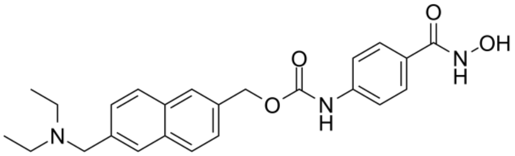
Repotrectinib	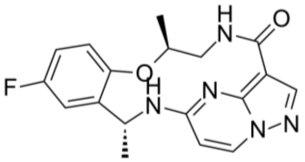
Zotiraciclib	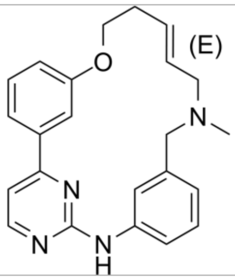
